# Editorial: The role of nutrition in enhancing surgical recovery and outcomes

**DOI:** 10.3389/fnut.2026.1878589

**Published:** 2026-06-10

**Authors:** Ann-Kathrin Lederer, Vasilios Pergialiotis, John M. Le

**Affiliations:** 1 Department of General and Visceral Surgery, Ulm University Hospital, Ulm, Germany; 2 Department of Internal Medicine II, Center for Complementary Medicine, Medical Center-University of Freiburg, Faculty of Medicine, University of Freiburg, Freiburg, Germany; 3 First Department of Obstetrics and Gynecology, Alexandra Hospital, National and Kapodistrian University of Athens, Athens, Greece; 4 Department of Oral and Maxillofacial Surgery, University of Alabama at Birmingham, Birmingham, AL, United States

**Keywords:** enhanced recovery after surgery, malnutrition, nutrition therapy, perioperative care, postoperative complications, sarcopenia, wound healing

Surgical recovery is a multidimensional process in which technical excellence alone is not enough to optimize outcomes. Nutritional status before, during, and after surgery influences immune competence, wound healing, muscle preservation, metabolic resilience, and the capacity to recover functional independence. This Research Topic brings together clinical trials, observational studies, systematic reviews, and case reports that collectively underscore nutrition as a core component of perioperative care.

## Identifying nutritional risk: the necessity for standardized screening

Bobos et al. examined the concurrent validity of the Global Leadership Initiative on Malnutrition (GLIM) criteria in 105 adults undergoing elective colorectal cancer surgery, comparing all 21 phenotypic-etiologic combinations against the modified Subjective Global Assessment (mSGA) as a reference standard. While aggregate GLIM demonstrated high sensitivity (97%) but modest specificity (64%), four specific combinations—P1EA, P12EA, P13EA, and P123EA—showed meaningful concordance with mSGA, suggesting that a targeted subset of GLIM criteria may offer a pragmatic and sufficiently accurate approach to identify preoperative malnutrition in this population.

Dang et al. conducted a prospective observational study including three widely used nutritional indices—the Mini Nutritional Assessment (MNA), the Prognostic Nutritional Index (PNI), and the Geriatric Nutritional Risk Index (GNRI) —for predicting postoperative delirium (POD) in 303 elderly patients undergoing non-cardiac surgery. MNA emerged as the superior predictor (AUC = 0.741), outperforming both PNI and GNRI. A combined model incorporating age, C-reactive protein (CRP), and MNA achieved an AUC of 0.810, providing a clinically actionable tool for preoperative risk stratification of POD.

Li et al. investigated the GNRI as a predictor of perioperative cardiovascular events (PCEs) in a multicenter retrospective analysis including 7,272 older patients with coronary artery disease undergoing non-cardiac surgery. GNRI demonstrated an independent and inverse linear association with PCEs, with at-risk patients (GNRI < 98) facing a significantly higher rate of PCEs. A combined scoring model integrating GNRI with the revised cardiac risk index (RCRI) significantly outperformed either index alone (AUC 0.768 vs. 0.694 for RCRI alone), suggesting that nutritional status adds prognostic information beyond traditional cardiac risk factors. These findings advocate for the routine incorporation of GNRI into preoperative cardiovascular assessment protocols, particularly for older surgical patients.

Wang T. et al. demonstrated that a low prognostic nutritional index (PNI) was an independent predictor of postoperative pneumonia following hematoma evacuation for intracerebral hemorrhage, with a combined predictive model incorporating low PNI, low admission score of Glasgow Coma Scale, obstructive lung disease, hypoproteinemia, and tracheotomy achieving an AUC of 0.87. In a markedly different population, Gu et al. evaluated PNI in 3,082 neonates and infants undergoing cardiac surgery, establishing it as an independent predictor of in-hospital mortality (AUC = 0.745).

Beyond nutritional indices, body composition parameters derived from routine computed tomography imaging, as well as laboratory-based markers, have emerged as powerful, objective surrogates of nutritional status. Zhu et al. demonstrated in a retrospective analysis of 200 patients with upper gastrointestinal perforation that low skeletal muscle reserve and high visceral fat area (VFA) independently predicted septic shock and ICU admission. Similarly, Xiang et al. identified the Controlling Nutritional Status (CONUT) score as an independent predictor of amputation risk in 387 patients with diabetic foot ulcers, with an AUC of 0.705. Xu B. et al. evaluated the impact of sarcopenia and treatment-related skeletal muscle loss during neoadjuvant immunochemotherapy in 272 patients with locally advanced esophageal squamous cell carcinoma. Sarcopenia and dynamic changes in skeletal muscle mass were assessed using computed tomography-based body composition analysis at the level of the third lumbar vertebra. While baseline sarcopenia was associated with lower pathological complete response rates, excessive skeletal muscle loss during treatment emerged as an independent predictor of poorer overall survival and was associated with increased hematologic toxicity, underscoring the importance of dynamic nutritional and body composition monitoring during oncological treatment.

## Prehabilitation and preoperative optimization

Recognition of nutritional risk is only the first step. The more clinically impactful question is whether preoperative nutritional optimization translates into improved surgical outcomes. Two randomized controlled trials in this collection provide compelling evidence supporting the beneficial impact of preoperative nutritional optimization on surgical outcomes, particularly when nutrition is embedded within multimodal prehabilitation programs. A further multicenter retrospective study investigated the significance of perioperative anemia for the development of postoperative delirium (POD) in older patients undergoing hip fracture surgery.

Mu et al. evaluated a 1-week multimodal prehabilitation program—integrating exercise, nutritional optimization, and psychological support—in 131 patients undergoing radical gastrectomy for gastric cancer. The prehabilitation group demonstrated a significantly lower 30-day complication rate (9% vs. 26%), earlier gastrointestinal recovery, faster postoperative mobilization, and a shorter hospital stay. The magnitude of benefit is notable despite the short duration of the intervention, suggesting that brief, structured prehabilitation programs can improve perioperative risk profiles in gastrointestinal oncology patients.

An et al. extended this approach to older adults, evaluating an multidisciplinary team-based perioperative management model in 115 patients aged 65 years or older undergoing major abdominal surgery. The intervention, which encompassed individualized exercise, nutritional support, psychological care, and post-discharge monitoring via wearable technology, reduced postoperative hospital stay by a median of 3.7 days and improved body composition outcomes including reduced weight loss and increased appendicular skeletal muscle mass index.

Xu X. et al. investigated in 1,694 older adults undergoing hip fracture surgery the association between perioperative anemia and POD. Severe anemia (hemoglobin < 75 g/L) was independently associated with an approximately three-fold higher POD risk, while lower perioperative hemoglobin levels mediated a substantial proportion of this effect. Given that anemia in older surgical patients is frequently linked to malnutrition, micronutrient deficiencies, and frailty, the findings further highlight the relevance of perioperative nutritional assessment and hemoglobin optimization in geriatric surgical care.

## Perioperative nutritional support: route, timing, and composition

Liu et al. conducted a bibliometric analysis of 269 publications on preoperative carbohydrate loading (PCL) spanning four decades, mapping the evolution of the field from early physiological validation studies to widespread clinical integration within enhanced recovery after surgery (ERAS) protocols. Their analysis identifies ongoing research frontiers including personalization of PCL regimens based on individual metabolic responses, harmonization of outcome measures, and integration of digital health technologies—highlighting that despite broad clinical adoption, important evidence gaps remain.

Wang J. et al. provided mechanistic insight into why early enteral nutrition (EEN) matters. The authors demonstrated in 745 patients after digestive surgery that intestinal barrier impairment, which was assessed via serum diamine oxidase, D-lactate, and lipopolysaccharide, was present in over 75% of postoperative patients and was independently associated with systemic inflammation and malnutrition. Interestingly, EEN was associated with significantly lower levels of intestinal barrier injury biomarkers compared to complete fasting, and 1 week of nutritional intervention improved intestinal permeability in the majority of patients.

Fu et al. corroborated the value of EEN in 138 patients undergoing minimally invasive surgery for intracranial aneurysms, demonstrating that EEN combined with enhanced nursing care improved mobilization, gastrointestinal recovery, nutritional and immune markers, and quality of life, compared to standard parenteral nutrition and conventional nursing care. The synergistic effect of nutritional and nursing interventions observed in this study echoes the broader theme of multimodal care pathways evident throughout this Research Topic.

Xu and Mao investigated the impact of a formulary prescription-based nutrition therapy protocol on parenteral nutrition practices and postoperative outcomes in surgical patients. Implementation of the protocol shifted practice from multi-bottle parenteral nutrition systems toward all-in-one admixtures and, more broadly, from total parenteral nutrition toward supplemental and total enteral nutrition. In patients with gastrointestinal cancers, this translated into significantly higher postoperative prealbumin levels and a shorter postoperative hospital stay, underscoring that system-level interventions to optimize nutritional prescribing practices can yield meaningful clinical benefits.

The impact of a structured postoperative nutritional protocol was also highlighted by Rossoni and Ribeiro, who reported excellent food tolerance and preserved intestinal function in a patient following transit bipartition.

At the level of specific nutritional components, Tang et al. reported that the combined postoperative administration of vitamin B1 and B12, but not either vitamin alone, significantly reduced time to first flatus after rectal cancer surgery in multivariate analysis, suggesting a synergistic effect that may operate through modulation of gut microbiota and autonomic nervous system function.

Sartini et al. provided a comprehensive overview of perioperative nutritional supplementation strategies including immunonutrition, probiotics, synbiotics, and protein supplementation, and their association with postoperative complications in a systematic review and meta-analysis of 39 studies. Immunonutrition was associated with a reduction in infectious complications (OR = 0.36) and showed a probable reduction in SSIs (OR = 0.35). The authors advocate for prioritization of immunonutrition in high-risk surgical settings based on current RCT evidence, while calling for standardization of outcome definitions and formulation protocols to facilitate future research.

## Special populations

Xu Y. et al. demonstrated that structured quantitative dietary guidance following transjugular intrahepatic portosystemic shunt (TIPS) placement in cirrhotic patients significantly reduced overall mortality (5.6% vs. 21.1%), liver-related mortality (1.9% vs. 15.8%), and hepatic encephalopathy occurrence (13.0% vs. 36.8%).

Finally, Shan et al. presented a case report illustrating the exceptional complexity of nutritional management in a patient with 96% total body surface area burns and acute kidney injury requiring 95 consecutive days of continuous renal replacement therapy, in whom a dynamically individualized nutritional strategy guided by indirect calorimetry and multidisciplinary collaboration was instrumental in achieving survival to discharge.

## Conclusions and future directions

The studies included in this Research Topic reinforce the concept that perioperative nutrition is not an adjunctive intervention, but a fundamental determinant of surgical outcomes. Across diverse surgical populations and clinical settings, nutritional status consistently emerged as a predictor of postoperative complications, functional recovery, treatment tolerance, and survival. Importantly, the evidence presented extends beyond traditional malnutrition screening, highlighting the growing relevance of objective body composition analysis, dynamic metabolic monitoring, and integrated nutritional risk assessment tools.

The contributions further demonstrate that nutritional interventions are most effective when embedded within multimodal perioperative care pathways. A recurring theme throughout this collection is the need to move from static and isolated nutritional assessments toward continuous, personalized, and multidisciplinary nutritional care integrated across the entire surgical trajectory. Several studies emphasized the importance of tailoring nutritional therapy to specific patient populations, including older adults, oncology patients, critically ill individuals, and patients with chronic liver disease or severe burns.

